# Bio-artificial pleura using an autologous dermal fibroblast sheet

**DOI:** 10.1038/s41536-017-0031-2

**Published:** 2017-10-11

**Authors:** Masato Kanzaki, Ryo Takagi, Kaoru Washio, Mami Kokubo, Masayuki Yamato

**Affiliations:** 1 The Department of Surgery I, Tokyo Women’s Medical University, Tokyo, Japan; 2 Institute of Advanced Biomedical Engineering and Science, Tokyo Women’s Medical University, Tokyo, Japan

## Abstract

Air leaks (ALs) are observed after pulmonary resections, and without proper treatment, can produce severe complications. AL prevention is a critical objective for managing patients after pulmonary resection. This study applied autologous dermal fibroblast sheets (DFS) to close ALs. For sealing ALs in a 44-year-old male human patient with multiple bullae, a 5 × 15-mm section of skin was surgically excised. From this skin specimen, primary dermal fibroblasts were isolated and cultured for 4 weeks to produce DFSs that were harvested after a 10-day culture. ALs were completely sealed using surgical placement of these autologous DFSs. DFS were found to be a durable long-term AL sealant, exhibiting requisite flexibility, elasticity, durability, biocompatibility, and usability, resulting reliable AL closure. DFS should prove to be an extremely useful tissue-engineered pleura substitute.

Air leaks (ALs) are frequently observed after pulmonary resections, especially video-assisted thoracoscopic surgeries (VATS). While often non-life-threatening, without proper treatment, ALs can progress to more severe complications.^1,2^ Based on the STS National Database (General Thoracic Surgery Database), Attaar et al. reported an incidence rate of prolonged air leak (PAL) of 8.6%.^3^ Furthermore, patients with PALs have significantly prolonged median lengths of hospital stays with the higher rates of in-hospital mortality.

The authors previously reported a novel method of cell-sheet engineering of lung AL sealants using DFSs harvested from temperature-responsive culture dishes (TCDs).^4,5^ This study demonstrates the first known application of autologous DFS for closing a human pleural defect.

A 44-year-old male Japanese patient presenting with left chest pain and shortness of breath upon exercise over the preceding month was referred to the authors’ hospital with an infected bulla. Chest computed tomography (CT) revealed multiple bullae in his left upper lobe, and one of these bullae contained fluid (Fig. 1a). A 5 × 15-mm surgical skin excision harvested from the anticipated surgical incision site (Fig. 1b) was used to source primary human dermal fibroblasts were cultured on tissue-culture dishes for 4 weeks (Fig. 1c). Then, culture-expanded fibroblasts were seeded onto 35-mm TCDs, and confluent DFSs were harvested without enzyme treatment after a 10-day culture (Fig. 1d). On the day prior to surgery, freshly prepared DFSs were subjected to various quality assessments, including sterility, mycoplasma, and endotoxin tests. Simultaneously, DFSs were cultured for investigating the number, viability, and purity of cells by measuring vimentin-positive-cell rate by flow cytometry, and DFSs found to contain 99.8% human fibroblasts. The patient underwent VATS bullectomy with a stapler. AL from the resection stump was confirmed by observing air bubbles upon submergence with saline. All ALs were completely sealed using autologous DFSs, and a water seal test confirmed stable AL closure against up to 25 cm H_2_O (Fig. 1e). Using a digital drainage system, no AL was detected after surgery. The postoperative course was uncomplicated. CT at one month post-surgery revealed no air space (Fig. 1f). No event was observed for 7 months after surgery, and the patient was found to be in a healthy condition.

**Fig. 1:**
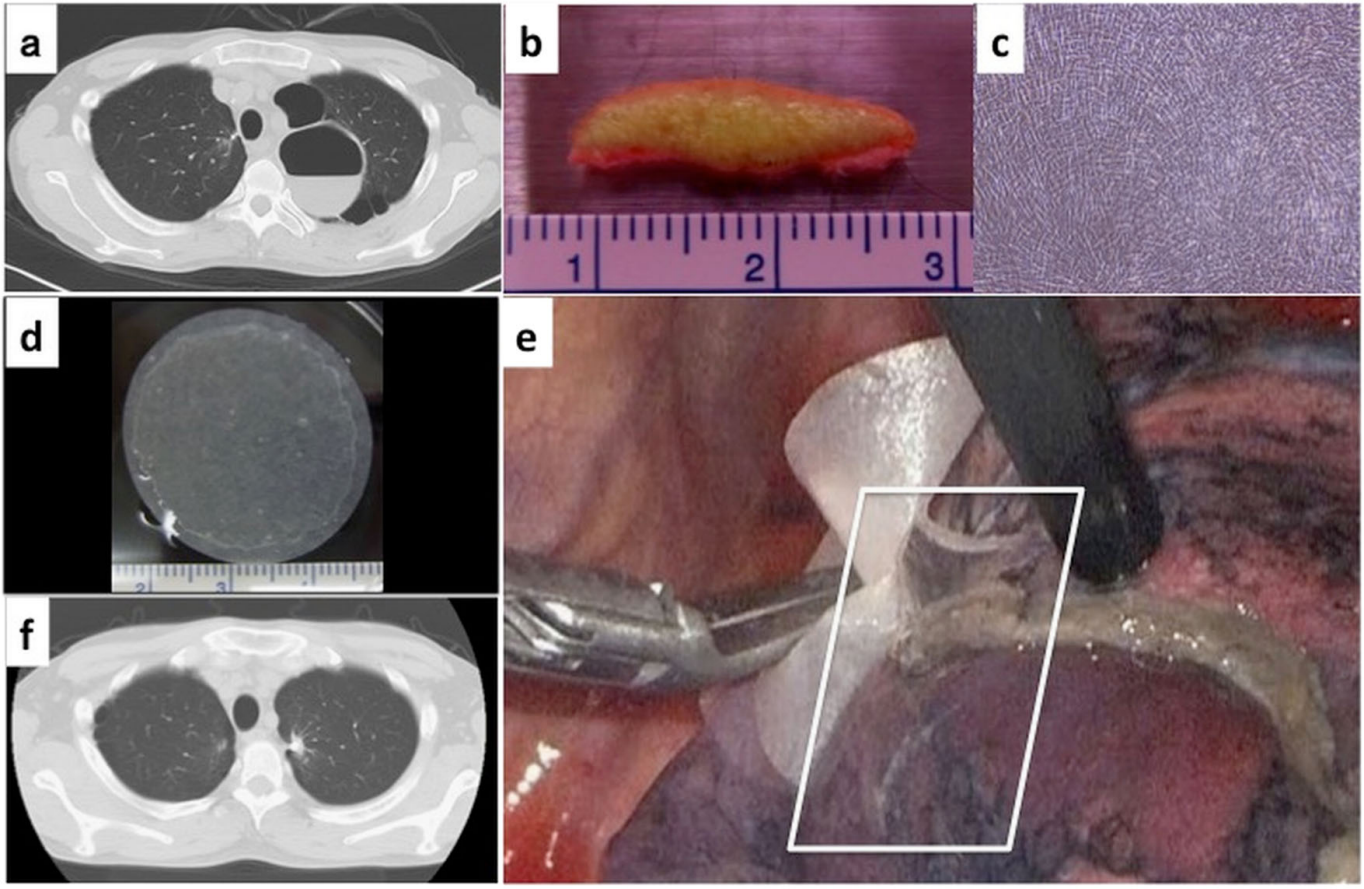
**a** Chest CT revealed multiple bullae in patient’s left upper lobe. **b** A 5 × 15-mm piece of patient skin was surgically excised from the planned surgical location of the dermal incision for the surgical repair. **c** Phase contrast micrograph of autologous primary dermal fibroblasts cultured for 4 weeks. **d** Transplantable dermal fibroblast sheet (DFS) harvested after 10-day culture. **e** All intraoperative ALs were completely sealed by transplanted DFSs (white rectangle). **f** Patient chest CT at one month post-surgery showing no observable air space

The methods were performed in accordance with relevant guidelines and regulations and approved by the ethics committee at Tokyo Women’s Medical University and by the Institutional Review Board for Clinical Research at Osaka University. Oral and written informed consents were obtained from the patient. This study was performed according to the Guidelines on clinical research using human stem cells established by the Ministry of Health, Labour, and Welfare, Japan, and registered with the University hospital Medical Information Network (UMIN) Clinical Trials Registry as No. UMIN000022554.

PALs after lung resection have negative consequences on overall morbidity, cost, and hospitalization. Intraoperative ALs should be avoided using careful surgical techniques. Various methods are currently applied to intraoperative AL closure. Especially, fibrin glue (FG)-based techniques with FG with sheet materials are preferred to close AL in Japan. However, due to the inflexibility of materials, FG-techniques give unsuccessful results. For treating recurred PALs, pleurodesis is performed with antibiotic, etc. Having various extracellular-matrix proteins, fibroblast sheets adhere to the pleural surface and close postoperative AL. Autologous DFS was used as a durable long-term AL sealant that was flexible enough to move responding to the dynamic movement of lung during respiration. This study validated the efficacy of DFS as bio-artificial pleura, which sealed AL.
